# Comparative Hydrodynamic Assessment of Twill-Woven Feed Spacers in Flat-Sheet Ultrafiltration Membrane Cassettes: Balancing Hydraulic Resistance and Near-Membrane Flow Enhancement

**DOI:** 10.3390/membranes16100329

**Published:** 2026-09-30

**Authors:** Shu Li, Hongbo Huang, Dongzhu Wu, Junteng Liu

**Affiliations:** 1 College of Chemical Engineering, Beijing University of Chemical Technology, Chaoyang Campus, Beijing 100029, China; 2025210006@buct.edu.cn (S.L.); 2024010294@buct.edu.cn (H.H.); 2 Hangzhou Horizone Technologies Co., Ltd., Hangzhou 310018, China

**Keywords:** feed spacer, twill-woven spacer, ultrafiltration, computational fluid dynamics, hydrodynamic assessment, wall shear stress

## Abstract

Feed spacers can promote membrane-side transport in flat-sheet ultrafiltration channels, but the accompanying hydraulic resistance increases pumping demand. This study combined pure-water pressure-drop measurements with transient three-dimensional ANSYS Fluent simulations to compare a commercial 2/1 twill-woven spacer (T-Base) and 16 systematically varied candidate configurations. A geometry-resolved finite-volume model was evaluated through computational-domain, mesh, and time-step sensitivity assessments; the predicted pressure gradients agreed with the measurements with a mean relative deviation below 4%. The methodological novelty lies in integrating pressure-normalized near-wall efficacy, bilateral wall shear stress balance, and equal-area spatial sampling within a geometry-resolved, multi-indicator framework for comparing spacer configurations with different periodic lengths and mesh densities. At a 60° low attack angle and a superficial inlet velocity of 0.0551 m·s^−1^, T3823 (filament spacing 0.38 mm, filament diameter 0.23 mm, spacer thickness 0.44 mm) provided the most favorable hydrodynamic trade-off among the 16 candidates. It produced a near-membrane velocity of 0.0220 m·s^−1^ and a pressure gradient of 53.5 kPa·m^−1^; its pressure-normalized near-wall efficacy was 1.23 relative to T-Base. T3823 also reduced bilateral membrane shear deviation from 24.91% to 8.30% and near-wall velocity dispersion by 50.7%. Overall, the results highlight the value of coordinated spacer-geometry optimization for balancing near-membrane flow enhancement and hydraulic resistance, with T3823 emerging as the most promising configuration among the candidates evaluated.

## 1. Introduction

Membrane separation technology has been widely applied in water treatment and bioseparation for efficient component separation in mixed systems. Among various membrane processes, ultrafiltration plays a central role for protein retention because of its characteristic pore-size range. Flat-sheet ultrafiltration cassettes are simple, compact and easy to operate, and are therefore commonly used for laboratory-scale tangential flow filtration processes. Feed spacers in flat-sheet ultrafiltration modules not only support the membrane layers and form flow channels, but also effectively intensify fluid disturbance [1]. Spacer geometry causes the flow to undergo such as contraction, expansion, flow deflection and local acceleration, which reduces low-velocity regions in the channel and facilitates the boundary-layer renewal near the membrane surface.

However, when flow is through spacer-filled channels, considerable pressure drop occurs owing to the combined effects of viscous drag and form drag. Excessive axial pressure drop not only increases the system’s operating energy demand, but also induces an axial gradient in transmembrane pressure, resulting in non-uniform flux distribution over the membrane surface and increased foulant accumulation in flow dead zones [2,3]. Meanwhile, under high-pressure-drop operating conditions, the pump outlet pressure has to be increased to maintain the target level of crossflow-induced shear, which raises the risk of shear-induced damage to biomolecules such as proteins and peptides [4]. Therefore, feed-spacer design must strike a balance between enhancing flow disturbance near the membrane surface and minimizing hydraulic resistance.

Recent advances in membrane-based separation further show that this hydrodynamic trade-off is strongly product- and process-dependent. Hybrid electrokinetic tangential flow filtration and tangential flow filtration coupled with size-exclusion chromatography have improved extracellular-vesicle recovery, impurity removal, and scalability by combining tangential surface sweeping with complementary separation mechanisms [5,6]. Tangential flow filtration has also enabled continuous dewatering of crystallized proteins, although maintaining a stable flux depends on wall shear rate, fiber length and the prevention of particle breakage or channel blockage [7]. Cascade tangential flow filtration can fractionate lignin streams over a wide molecular-weight range, but the required transmembrane pressure and crossflow velocity directly affect recovery and cost [8]. Studies of flat-sheet membrane contactors likewise show that increasing the liquid flow rate can reduce liquid-side mass-transfer resistance while simultaneously altering membrane stability [9]. At the membrane-material level, next-generation ultrafiltration membranes may offer improved selectivity and antifouling behavior, yet the practical performance of protein concentration processes remains governed by boundary-layer resistance and the gel layer formed by retained proteins [10]. These findings emphasize that spacer and channel design must be evaluated in terms of product quality, fouling propensity, throughput and energy consumption rather than on the basis of permeate flux alone.

Computational fluid dynamics (CFD) has consequently become a central tool for resolving the coupled momentum and mass-transfer processes that cannot be inferred from channel-averaged quantities. Reviews of osmotic membrane systems and membrane distillation show that CFD can reveal spacer-induced mixing, polarization and local transport, while also identifying persistent limitations in meshing of complex geometries, fouling representation and multi-scale validation [11]. Data-driven and machine-learning models can accelerate module-level prediction, but their reliability remains conditional on the quantity and quality of the training data [12]. CFD-derived correlations for concentration polarization are more consistent than competing pressure-drop correlations [2]. Mathematical models of flat-sheet forward-osmosis modules [13] and gas-separation modules [14] further show that membrane length, channel height and geometric non-uniformity alter boundary-layer development, separation performance and required membrane area. ANSYS Fluent 2022 R2(ANSYS, Inc., Canonsburg, PA, USA), the finite-volume solver used here, implements the incompressible conservation equations and provides benchmark verification cases for fluid-flow solutions [15,16]. The studies have shown that Fluent-based predictions can agree with established correlations and experimental flow quantities when the governing formulation, grid resolution, and temporal resolution are appropriately verified [17,18]. These studies also show that model validity remains problem- and regime-dependent. Geometry-resolved CFD is therefore suitable for comparing spacer configurations with different local constrictions and periodic lengths, provided that the numerical solution is independently verified. Accordingly, the present model was evaluated through computational-domain, mesh, and time-step sensitivity analyses and validation against the measured pure-water pressure gradients.

Parametric spacer studies indicate that spacer performance cannot be optimized reliably by varying a single geometric parameter in isolation. For woven and overlapped spacers, filament pitch, flow attack angle, and Reynolds number jointly govern secondary-flow development, mass-transfer enhancement, and specific power consumption [19]. Spacer configurations with inclined oval filaments can reduce pressure loss and dead zones, but the associated concentration-polarization response differs from that of circular spacers [20]. Additive manufacturing has expanded the accessible design space [21]: a 3D-printed column spacer reduced pressure drop and specific energy consumption while shortening stagnant regions [22], whereas multi-layer spacers increased mass transfer but imposed a substantially larger friction factor [23]. Channel porosity is likewise non-monotonic with respect to biofouling, because both excessively open low-shear meshes and highly obstructed channels can promote biomass accumulation through different mechanisms [24]. Non-uniform filament cross-sections can reduce pressure loss, but may also decrease membrane wall shear stress and permeate flux [25]. Channel-gap reduction changes pressure loss, wall shear and mass transfer even when the nominal spacer topology is unchanged [26]. More recent CFD simulations, particle-transport analyses, and filtration experiments show that carefully designed non-woven or pillar-type spacers can reduce stagnant zones, fouling and energy consumption while increasing permeate flux [27]. Together, these studies demonstrate that hydraulic resistance, near-wall momentum transfer, flow uniformity and manufacturability must be treated as coupled design objectives.

The relation between local hydrodynamics and fouling is similarly more complex than a simple assumption that higher mean shear is always beneficial. Patterned membrane surfaces can generate vortices that return deposited particles to the bulk stream [28], while electro-osmotic forcing can intensify near-wall mixing and vortex shedding with a lower pumping-energy proxy [29]. Dynamic filtration using a rotating membrane [30] or rotating spacer can generate additional shear stresses and lift forces without relying exclusively on a higher axial cross-flow rate. Nevertheless, shear-controlled cake removal is effective only within particular flux and foulant-concentration ranges [31]. A wavy flat-sheet membrane bioreactor improved the uniformity of bubble distribution and wall shear while reducing fouling resistance [32], whereas a comparison of hollow-fiber and spacer-filled flat-sheet modules showed that spacer-generated dead zones can change both reversible and irreversible fouling pathways [33]. Recent work further demonstrates that the preferred flow field differs for scaling and biofouling: scaling mitigation benefits from enhanced shear and solute mass transfer, whereas biofouling control depends more strongly on minimizing persistent low-velocity regions [34]. Fluid–structure interaction adds another layer, because membrane deformation can reshape near-surface shear, delay fouling and alter flux decline [35]. Thus, spatial distributions of velocity and wall shear stress, rather than their surface averages alone, are required to evaluate the membrane environment created by a spacer.

Evidence from related channel-flow systems reinforces this need for spatially resolved assessment. In direct-methanol fuel cells, flow-field pattern, channel width and open ratio govern pressure loss, reactant distribution and product removal, so designs that improve one transport limitation may intensify another [36]. In water-distribution cleaning, CFD coupled with field measurements has shown that wall shear stress is non-uniform and that maintenance effectiveness depends on both the magnitude and spatial persistence of the imposed stress [37]. Although these systems differ from membrane cassettes, they demonstrate a transferable principle: bulk flow rate or total pressure drop cannot by itself describe how a three-dimensional internal structure redistributes momentum at a functional surface.

Despite these advances, important gaps remain for flat-sheet ultrafiltration cassettes containing 2/1 twill-woven feed spacers. Most recent comparative studies concern spiral-wound reverse-osmosis or nanofiltration modules, non-woven spacers, idealized node-and-filament structures, or alternative dynamic-filtration devices. A geometry-resolved study of a commercial protein-ultrafiltration cassette has recently shown that membrane imprinting can reduce the effective channel height, strengthen recirculation, increase pressure drop and produce differences of up to 25% between the average shear stresses on the two membrane surfaces [1]. This finding is consistent with the strong sensitivity of pressure loss and wall shear to channel-gap reduction [26], but the coupled effects of filament spacing and the filament-diameter/spacer-thickness level in twill-woven structures remain insufficiently resolved. In addition, direct comparison of raw CFD nodes across geometries with different periodic lengths, void volumes and mesh densities can introduce unequal spatial weighting, obscuring differences in near-wall flow uniformity and bilateral membrane exposure.

Accordingly, this study combines pure-water pressure-drop experiments with transient laminar three-dimensional CFD to compare a commercial twill-woven spacer with 16 systematically varied candidate geometries at a fixed 60° flow attack angle. Filament spacing and the coupled filament-diameter/spacer-thickness level are varied, while domain-length, mesh and time-step sensitivity are assessed before cross-configuration comparison. The methodological contribution of this study is a geometry-resolved, multi-indicator framework that combines pressure gradient, average near-wall velocity, membrane wall shear stress, a pressure-normalized near-wall efficacy index, a bilateral wall-shear-stress imbalance index, and equal-area normalized spatial sampling. The objective is to screen for a candidate that converts hydraulic expenditure into effective and spatially balanced near-membrane transport within the investigated design space.

## 2. Materials and Methods

### 2.1. Spacer Geometry, Channel Porosity, and Computational Domain

**Figure 1:**
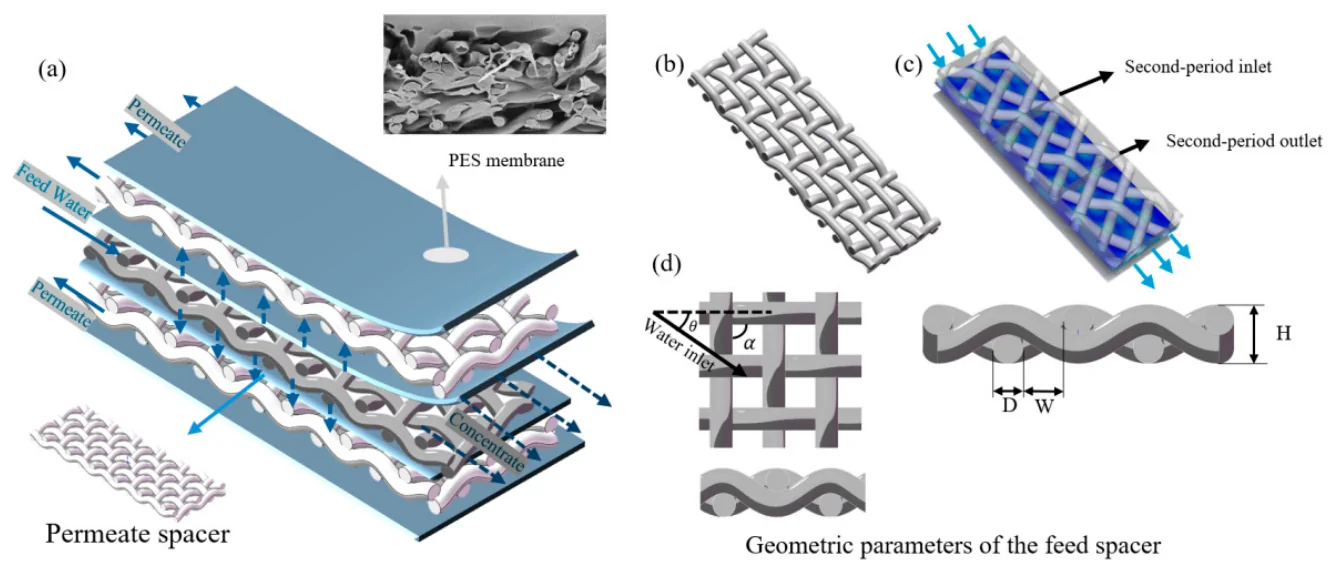
Schematic illustration of the ultrafiltration membrane cassette, twill-woven feed spacer, and computational domain: (**a**) spacer-filled membrane cassette and filtration pathways; (**b**) representative twill-woven feed spacer; (**c**) three-period computational fluid domain with the second-period inlet and outlet; and (**d**) definition of feed-spacer geometric parameters. The schematic and model components were prepared by the authors using Microsoft PowerPoint for Microsoft 365 (Microsoft Corporation, Redmond, WA, USA) and SOLIDWORKS 2024(Dassault Systèmes SolidWorks Corp., Waltham, MA, USA). No generative AI was used to create or modify Figure 1.

The flat-sheet ultrafiltration membrane module adopted in this study is assembled by alternately arranging membrane sheets and spacers, and its overall structure is shown in Figure 1a Polyethersulfone (PES) ultrafiltration membranes were used in the experiments. Feed spacers are sandwiched between two adjacent PES membrane sheets to form feed channels. After entering the spacer-filled feed channel, the feed solution flows tangentially across the membrane surface. Part of the feed permeates through the PES membrane and is collected as permeate via the permeate-side spacer, while the remaining feed exits the channel as retentate. Figure 1a illustrates the complete filtration pathway of the physical membrane module. The established CFD numerical model only analyzes the hydrodynamic characteristics on the feed side, and the transmembrane permeation process is not explicitly solved.

As shown in Figure 1b, the feed spacer features a 2/1 twill-weave structure. In each repeating unit, one filament passes over two successive interlaced filaments and then under the next filament. The gradual offset of this over–under interlacing sequence between adjacent filaments forms the characteristic diagonal twill pattern. In this work, the geometric parameters of the spacer are characterized by filament spacing W, filament diameter D, spacer thickness H, filament crossing angle α, and flow attack angle β, as illustrated in Figure 1d. The filament intersection angle is fixed at α = 90° for all cases, where β denotes the angle between the main flow direction and the orientation of the reference filament.

A commercial twill-woven spacer was selected as the baseline configuration (denoted as T-Base-), with geometric parameters of W = 0.297 mm, D = 0.215 mm, and H = 0.42 mm. Based on this baseline, a series of candidate spacers were constructed by varying the filament spacing W together with the coupled D–H parameter set. The parameters of all simulation cases are summarized in Table 1. The investigated filament spacing W values are 0.28, 0.31, 0.35, and 0.38 mm, with corresponding filament diameter D of 0.20, 0.23, 0.26, and 0.30 mm and spacer thickness H of 0.38, 0.44, 0.50, and 0.58 mm. Since D and H vary synchronously, they are treated as one coupled geometric parameter group in this work, and their individual effects are not separately interpreted.

To quantitatively characterize the volume fraction available for fluid flow within the feed channel, the channel porosity was calculated based on the reconstructed three-dimensional geometric model, as given below:

(1) $$\varepsilon =\frac{V_{f}}{V_{ch}}=1-\frac{V_{sp}}{V_{ch}},$$

where ε is the channel porosity, $V_{f}$ is the fluid-domain volume, $V_{sp}$ is the spacer volume, and $V_{ch}$ is the volume of the rectangular channel envelope before subtracting the spacer-solid volume. The channel-envelope volume, $V_{ch}$, was calculated from the geometric dimensions of the computational domain:

(2) $$V_{ch}=L_{x}L_{y}L_{z}.$$

### 2.2. Computational Model and Numerical Verification

#### 2.2.1. Governing Equations and Solver

The feed-side hydrodynamics were simulated in ANSYS Fluent using a three-dimensional finite-volume, transient, pressure-based formulation with the laminar-flow model. Pure water was treated as an incompressible Newtonian fluid with constant thermophysical properties. The governing continuity and incompressible Navier–Stokes momentum equations are [15,16]:

(3) ∇⋅u=0

$$\rho (\frac{\partial u}{\partial t}+u\cdot \nabla u)=-\nabla p+\mu \nabla^{2}u,$$

Here, u is the velocity vector, p is pressure, t is time, ρ is density, and μ is dynamic viscosity. For configuration i, the Reynolds number was based on the superficial (empty-channel) inlet velocity and the empty rectangular-channel hydraulic diameter:

(4) $${Re}_{i}=\frac{D_{h,i}\rho u_{s}}{\mu }$$

(5) $$u_{s}=\frac{Q}{BH_{i}}$$

(6) $$D_{h,i}=\frac{2BH_{i}}{B+H_{i}}\approx 2H_{i}$$

In Equation (5), Q denotes volumetric flow rate, B is channel width, and H_i_ is the channel height of configuration i. Because B ≫ H_i_, Equation (6) reduces to $D_{h,i}\approx 2H_{i}$. The 16-geometry comparison used u_s_ = 0.0551 m·s^−1^ and pure-water properties at 20 °C (ρ = 998.2 kg·m^−3^ and μ = 1.002 × 10^−3^ Pa·s). The resulting Reynolds numbers were approximately 41.7 for T2820, T3120, T3520 and T3820 (H = 0.38 mm); 48.3 for T2823, T3123, T3523, and T3823 (H = 0.44 mm); 54.9 for T2826, T3126, T3526, and T3826 (H = 0.50 mm) and 63.7 for T2830, T3130, T3530, and T3830 (H = 0.58 mm). For T-Base, the 350–540 mL·min^−1^ validation sweep corresponded to Re = 40.3–62.2 under the same convention. These Reynolds numbers fall within the laminar regime and therefore support the use of the laminar-flow model.

#### 2.2.2. Computational Domain Assessment

To determine an appropriate streamwise domain length and mitigate the interference of inlet/outlet development effects on the flow field, a domain length sensitivity analysis was conducted using the T-Base baseline geometry. Four computational domains were constructed, comprising 1–5 consecutive periodic units, respectively. For each domain, the pressure drop across each individual periodic unit was extracted and normalized by the corresponding unit length to obtain the pressure drop per unit length, which served as the indicator for assessing whether the flow reached a periodically fully developed state. A domain length was deemed sufficient when the relative deviation in pressure drop per unit length between adjacent domains was less than 5%, indicating that inlet/outlet end effects were negligible.

**Figure 2:**
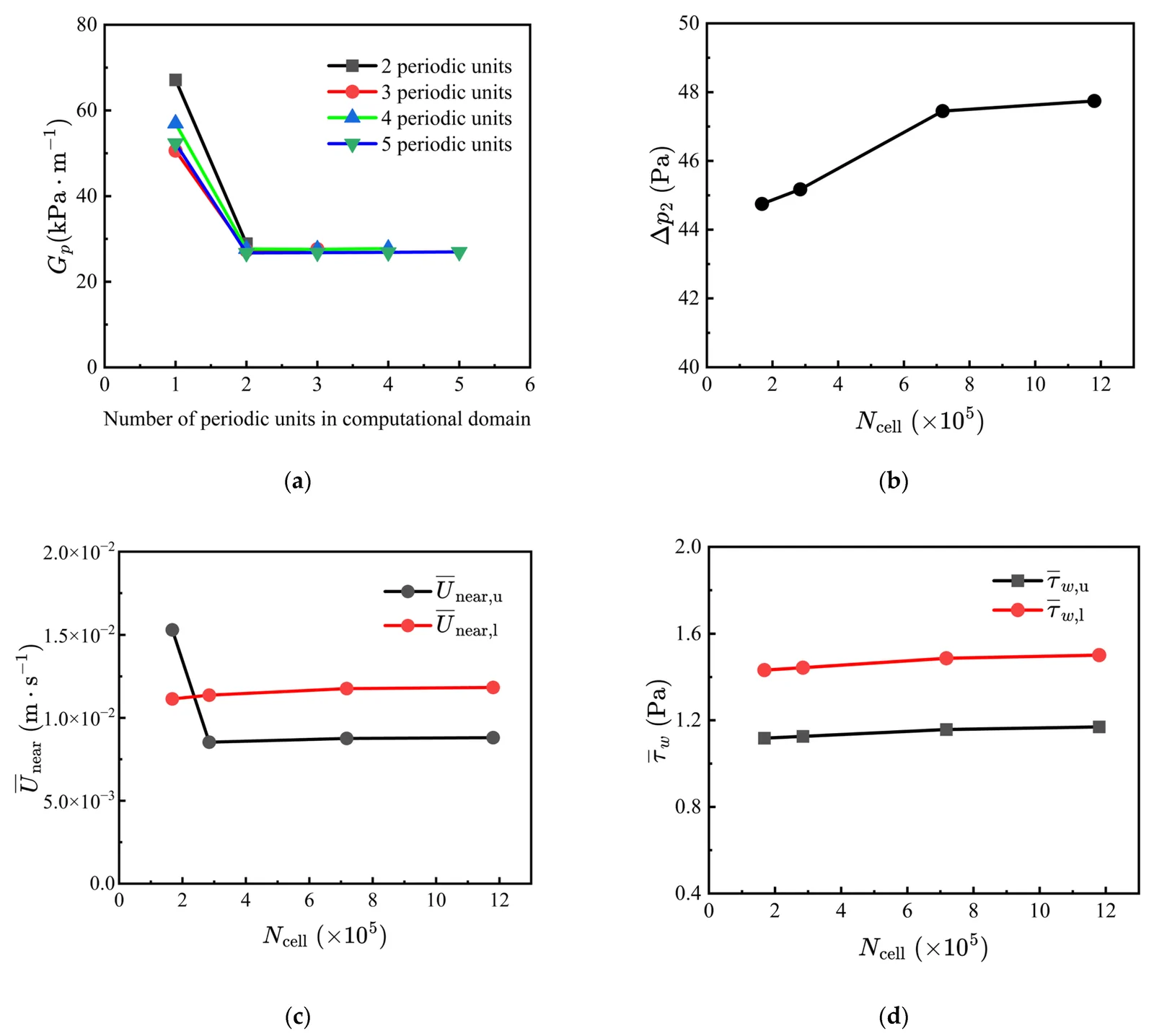
Numerical sensitivity assessment for the T-Base spacer in ANSYS Fluent: (**a**) pressure drop per unit length within computational domains containing two to five periodic units; mesh sensitivity of (**b**) the pressure drop across the representative second periodic unit, $\Delta p_{2}$, (**c**) upper- and lower-side average near-wall velocities, ${Ū}_{near,u}$ and ${Ū}_{near,l}$, and (**d**) upper- and lower-membrane average wall shear stresses, ${\bar{\tau }}_{w,u}$ and ${\bar{\tau }}_{w,l}$.

As shown in Figure 2a, the pressure drop per unit length across the first periodic unit was significantly higher than that across the subsequent units, indicating pronounced flow development effects at the inlet. From the second periodic unit onward, the pressure drop per unit length stabilized, with a relative deviation of less than 5% between adjacent units. These results demonstrate that the inlet development effect was primarily confined to the first periodic unit, and a periodically fully developed flow was established from the second unit onward.

Based on the above domain length sensitivity assessment, a computational domain comprising three consecutive periodic units was adopted for all subsequent numerical simulations in this study, as illustrated in Figure 1c. The first unit served as the inlet development section, the second unit was selected as the representative unit for hydrodynamic characterization, and the third unit functioned as the outlet buffer section to mitigate the upstream influence of the outlet boundary condition on the representative unit. The inlet and outlet of this representative unit were defined as the “second-period inlet” and “second-period outlet,” respectively. The extraction and evaluation of hydrodynamic parameters, including pressure drop, near-wall velocity, and wall shear stress, were performed within this representative periodic unit.

#### 2.2.3. Mesh Independence and Convergence Assessment

For the feed channel equipped with spacers, a Poly-Hexcore hybrid meshing strategy was employed to ensure good grid adaptability in regions with complex spacer geometry while maintaining high computational efficiency in the bulk flow region. Five inflation layers were applied near the upper and lower membrane walls to accurately resolve the near-wall velocity gradient; the inflation growth rate was set to 1.2. Four progressively refined grids (denoted M0–M3) were constructed, containing 168,192, 284,300, 717,843, and 1,184,521 mesh elements, respectively.

The grid independence was assessed using the following metrics: the pressure drop across the representative unit (${\Delta p}_{2}$), the area-averaged near-wall velocities at the upper and lower membrane walls, ${Ū}_{near,u}$ and ${Ū}_{near,l}$, and the corresponding wall shear stresses, ${\bar{\tau }}_{w,u}$ and ${\bar{\tau }}_{w,l}$, as depicted in Figure 2b–d.

All five monitored quantities stabilized as the mesh was progressively refined from M0 to M3. With the finest mesh, M3, as the reference, the relative deviations obtained with M2 were 0.56% and 0.58% in the upper and lower near-wall velocities, 1.06% and 0.96% in the corresponding wall shear stresses, and 0.62% in ${∆p}_{2}$; thus, all five deviations were below 1.1%. The concurrent stabilization of the pressure drop and near-wall quantities indicates practical grid independence across the tested mesh sequence. Mesh M2, containing 717,843 elements, was therefore selected for the remaining simulations because it provided sufficiently converged results at a lower computational cost than M3.

#### 2.2.4. Time-Step Sensitivity Assessment

The time step independence was assessed using three time steps of Δt = 0.020, 0.010, and 0.005 s (selected by a factor-of-two reduction), with a constant total physical time of 0.50 s, corresponding to 25, 50, and 100 time steps, respectively.

Table 2 summarizes the time-step sensitivity results. Relative to the reference solution obtained with Δt = 0.005 s, the maximum relative deviations across the monitored pressure drop, near-wall velocity, and wall shear stress were only 1.47 × 10^−5^% for Δt = 0.020 s and 1.98 × 10^−10^% for Δt = 0.010 s. These negligible deviations indicate that the solution was independent of time-step size over the investigated range. Accordingly, Δt = 0.010 s was selected for the subsequent transient simulations.

#### 2.2.5. Boundary Conditions, Initialization, and Solution Monitoring

A uniform superficial-velocity inlet boundary condition and a pressure-outlet boundary condition were employed, with a gauge pressure of 0 Pa at the outlet. The upper and lower membrane surfaces and all spacer-filament surfaces were treated as stationary no-slip walls. Before transient time marching, the velocity and pressure fields were initialized in ANSYS Fluent from the imposed inlet and outlet conditions. The simulations considered pure-water feed-side hydrodynamics, with the membrane surfaces treated as impermeable walls.

For comparison among the 16 candidate geometries, the superficial inlet velocity was fixed at $u_{av}=0.0551{m\cdot s}^{-1}$; for T-Base, this velocity corresponded to the experimental flow-rate condition of 400 mL·min^−1^. The maximum number of iterations per time step was 60. Pressure drop, near-wall velocity, wall shear stress, and inlet–outlet mass-flow balance were monitored. After the initial transient, these quantities became nearly time independent, and the resulting quasi-steady hydrodynamic field was used for cross-configuration comparisons.

### 2.3. Experimental Pressure-Drop Measurement

Pressure drop experiments were conducted using a closed-loop pure water system. After assembling the membrane module into the test cell, a calibrated torque wrench was used to uniformly tighten the module to a prescribed torque, ensuring consistent spacer compression and channel height across repeated tests. The experimental system consisted of a pure water reservoir, a peristaltic pump, a membrane module, connecting tubing, and high-precision pressure transducers installed at the inlet and outlet of the feed channel. The custom-built membrane cassette and hydraulic testing apparatus were supplied by Hangzhou Horizone Technologies Co., Ltd. (Hangzhou, China). The pressure drop was determined as the difference between the measured inlet and outlet pressures.

Prior to each experiment, the system was thoroughly flushed with pure water to remove residual impurities and trapped air. The permeate-side outlet was then closed to eliminate transmembrane permeation effects, ensuring that the measured pressure drop arose solely from the hydrodynamic resistance of the feed channel. The feed flow rate was sequentially adjusted to specified values using the peristaltic pump, and the inlet and outlet pressures were recorded once the system reached steady state. The measured pressure drop was normalized by the effective channel length to yield the pressure drop per unit length for comparison with the numerical results.

## 3. Results

### 3.1. Flow Attack Angle Selection and Model Validation

The flow attack angle β is defined as the angle between the inlet-flow direction and the alignment of the reference spacer filament. In practical flat-sheet ultrafiltration modules, β is jointly determined by spacer orientation and channel geometry. Measurements of the commercial module indicated a practical range of 30–60°; accordingly, three values of β = 30°, 45°, and 60° were evaluated both experimentally and numerically.

**Figure 3:**
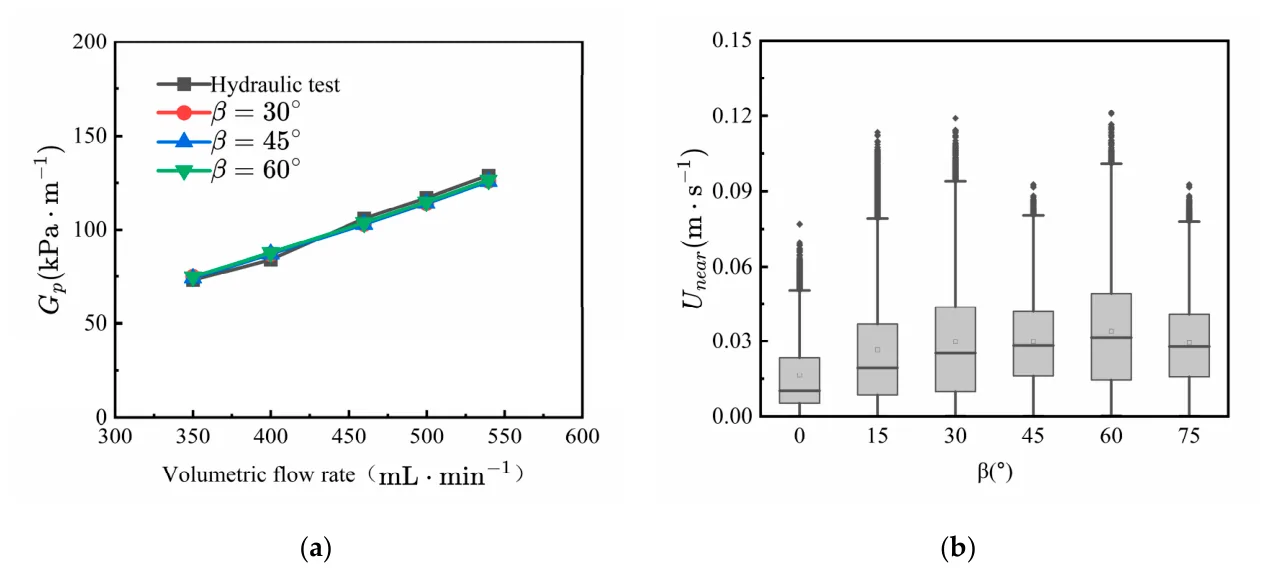
Effect of flow attack angle on spacer-filled-channel hydrodynamic: (**a**) measured and computational-fluid-dynamics-predicted pure-water pressure gradients at five flow rates; (**b**) near-membrane velocity distributions at flow attack angles of 0°~70°.

Pure-water pressure-drop experiments performed under matching operating conditions were used to evaluate the hydraulic-resistance prediction. As shown in Figure 3a, the CFD-predicted pressure gradients followed the measured trend over the investigated flow-rate range at each flow attack angle. The mean relative deviation between the measured and predicted pressure gradients was below 4%. This agreement supports the use of the model for comparative feed-channel hydrodynamics.

The flow attack angle had a limited effect on the overall pressure gradient but a stronger effect on the simulated near-membrane flow environment. As β increased, spacer-induced flow redirection increased the overall near-wall velocity, with the highest value among the tested angles occurring at β = 60°. This condition may favor membrane-surface renewal.

On the basis of the measured pressure-gradient agreement and the simulated near-membrane flow response, β = 60° was selected as the fixed flow attack angle for the subsequent comparison of spacer geometries.

### 3.2. Effects of Spacer Geometry on Hydrodynamic Performance

At β = 60°, a 4 × 4 full-factorial geometric comparison was performed using four filament spacings (W = 0.28, 0.31, 0.35, and 0.38 mm) and four coupled filament-diameter/spacer-thickness levels (D = 0.20, 0.23, 0.26, and 0.30 mm; H = 0.38, 0.44, 0.50, and 0.58 mm), yielding 16 candidate configurations.

**Figure 4:**
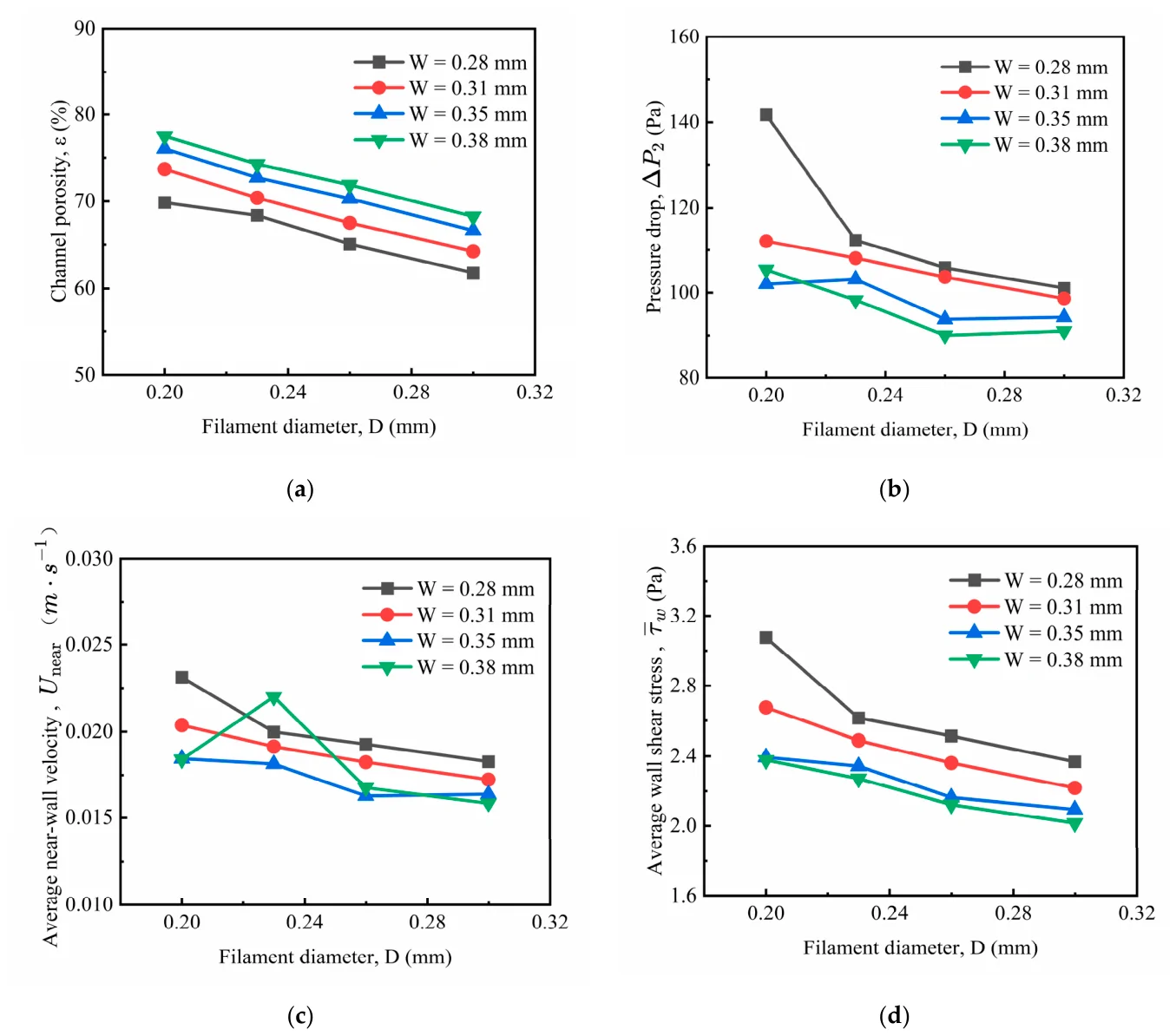
Effects of filament diameter and spacing on the hydrodynamic characteristics of twill-woven spacers: (**a**) channel porosity; (**b**) second-period pressure drop; (**c**) average near-membrane velocity; and (**d**) average membrane wall shear stress.

Figure 4a presents the variation in channel porosity with filament diameter under different filament spacings. For all spacer configurations, increasing the filament diameter increased the solid volume fraction of the spacer and reduced the effective flow space, thereby gradually decreasing the porosity. Meanwhile, increasing the filament spacing enlarged the flow cross-section and increased the overall porosity. The variation in filament diameter and spacing altered the degree of channel blockage, which further affected the pressure loss and near-membrane flow characteristics.

As shown in Figure 4b, the pressure drop across the second periodic unit was significantly influenced by the filament geometric parameters. At a fixed filament spacing, reducing the filament diameter generally decreased the pressure drop, as thinner filaments exerted weaker flow blockage and form drag. Meanwhile, increasing the filament spacing enlarged the flow cross-section and reduced the energy loss caused by flow contraction and expansion. Among all configurations, the combination of *W* = 0.28 mm and *D* = 0.30 mm exhibited the highest pressure drop, indicating that both the narrower channel and stronger blockage collectively increased the flow resistance.

Although reduced blockage generally lowered pressure loss, it also weakened the simulated near-membrane flow response. Figure 4c shows that area-averaged near-wall velocity varied non-monotonically with the coupled D–H level. For example, at W = 0.38 mm, D = 0.23 mm (with H = 0.44 mm) produced the highest near-wall velocity within the four tested levels. Similar within-range maxima occurred at D = 0.23–0.26 mm for the other spacing groups. Thus, moderate obstruction can promote local acceleration and redistribution, whereas a more open channel can reduce spacer-induced perturbation.

Figure 4d shows that area-averaged wall shear stress generally decreased as the coupled D–H level increased. The accompanying increase in the gap between the spacer apex and membrane surface displaced the high-velocity core away from the membrane and weakened the near-wall velocity gradient; larger filament spacing also reduced local perturbation. Higher wall shear can create hydrodynamic conditions that may assist surface renewal. The comparative assessment therefore considers shear together with pressure loss and spatial uniformity.

In summary, filament spacing and the paired combinations of filament diameter and spacer thickness jointly determine hydraulic resistance and the simulated near-membrane flow response. The following analysis therefore compares all 16 candidates using multiple hydrodynamic indicators rather than treating any one geometric variable or performance measure as independently optimal.

### 3.3. Hydrodynamic Trade-Off and Selection of Candidate Configurations

Changes in filament spacing and coupled D–H level affect both flow resistance and near-membrane motion. Because stronger spacer-induced perturbation can increase both quantities, the candidates were screened by jointly considering hydraulic expenditure, near-wall velocity, and wall shear rather than by maximizing a single response. All comparisons in this section are limited to the 16 geometries at β = 60° and us = 0.0551 m·s^−1^.

**Figure 5:**
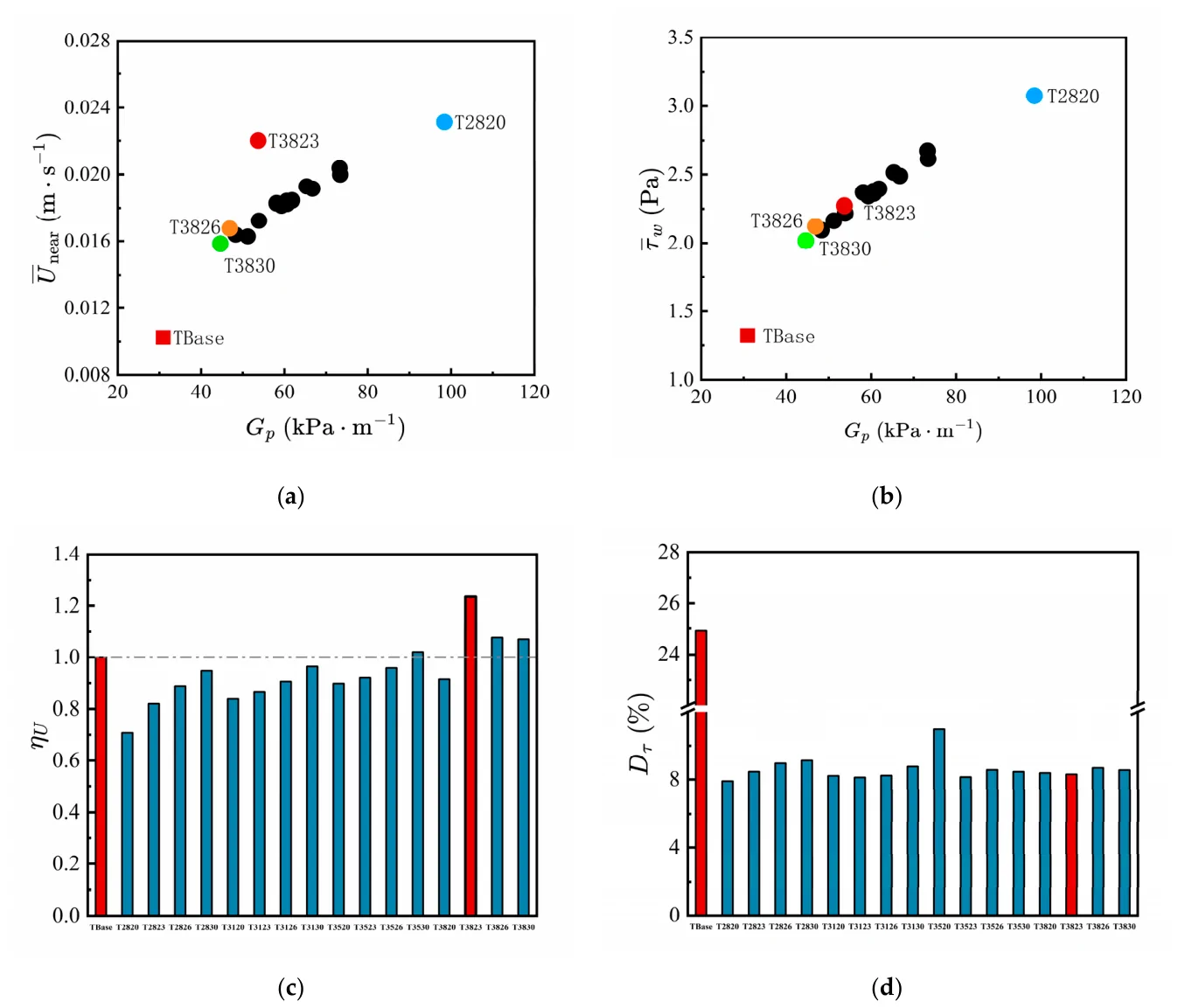
Multi-indicator hydrodynamic comparison of T-Base and 16 twill-woven spacer candidates at β = 60° and us = 0.0551 m·s^−1^: (**a**) area-average near-membrane velocity versus pressure gradient; (**b**) area-average membrane wall shear stress versus pressure gradient; (**c**) pressure-normalized near-wall efficacy relative to T-Base; and (**d**) bilateral membrane wall shear stress deviation.

Figure 5a relates pressure gradient G_p_ to area-averaged near-wall velocity for T-Base and the 16 candidates. All 16 candidate configurations exhibited a higher near-wall velocity than T-Base under the specified inlet condition, although the gain was not proportional to the pressure penalty. T2820 (W = 0.28 mm, D = 0.20 mm, H = 0.38 mm) produced the largest near-wall velocity (0.0231 m·s^−1^) but also a pressure gradient of 99.8 kPa·m^−1^. T3823 (W = 0.38 mm, D = 0.23 mm, H = 0.44 mm) produced a similar near-wall velocity (0.0220 m·s^−1^) with a lower pressure gradient of 53.5 kPa·m^−1^ and provided a more favorable balance between near-wall flow enhancement and hydraulic resistance.

Figure 5b relates area-averaged wall shear stress to pressure gradient. T2820 generated the highest shear but also incurred the largest hydraulic penalty among the configurations highlighted. T3823 maintained a comparatively high shear level at a lower pressure gradient.

To compare how effectively each configuration converted hydraulic expenditure into near-membrane motion, a pressure-normalized near-wall efficacy index η_U,i_ was defined relative to T-Base:

(7) $$\eta_{U,i}=\frac{{\overset{\text{-}}{Ū}}_{\mathrm{near},i}/G_{P,i}}{{\overset{\text{-}}{Ū}}_{\mathrm{near},\mathrm{TBase}}/G_{P,TBase}},$$

where ${\overset{\text{-}}{Ū}}_{\mathrm{near},i}$ is the area-averaged near-wall velocity of the evaluated configuration, and ${\overset{\text{-}}{Ū}}_{\mathrm{near},\mathrm{TBase}}$ is that of the baseline configuration T-Base. When $\eta_{U}$ > 1, the configuration possesses a stronger tangential flow enhancement capability at the membrane surface than the baseline.

As shown in Figure 5c, four of the 16 candidate configurations had $\eta_{U}$ > 1. T3823 had the largest value, $\eta_{U}$ = 1.23, corresponding to approximately 23% greater pressure-normalized near-wall efficacy than T-Base. This improvement refers to the normalized efficacy index, which incorporates both near-wall velocity and pressure gradient. The index therefore supports selection of T3823 as the strongest pressure-normalized candidate among the investigated geometries, while the remaining indicators are needed to assess shear magnitude and bilateral balance.

In addition to the average shear level, the consistency of the hydrodynamic environment between the upper and lower membrane surfaces is equally important for flat-sheet membrane modules. Since the spacer structure may induce different flow environments on the two membrane surfaces, a bilateral wall shear stress deviation index $D_{\tau }$ was defined:

(8) $$D_{\tau }=\frac{{\overset{\text{-}}{\bar{\tau }}}_{w,u}\text{-}{\overset{\text{-}}{\bar{\tau }}}_{w,l}}{{\overset{\text{-}}{\bar{\tau }}}_{w}}\times 100\%,$$

where ${\overset{\text{-}}{\bar{\tau }}}_{w,u}$ and ${\overset{\text{-}}{\bar{\tau }}}_{w,l}$ are the area-averaged wall shear stresses on the upper and lower membrane surfaces, respectively, and ${\overset{\text{-}}{\bar{\tau }}}_{w}$ is the overall average wall shear stress. A lower $D_{\tau }$ indicates a more balanced shear environment between the two membrane surfaces.

As shown in Figure 5d, all 16 candidate configurations exhibited a smaller difference in mean wall shear stress between the upper and lower membrane surfaces than T-Base. T3823 combined a low bilateral shear deviation of 8.30% with an area-averaged wall shear stress of 2.27 Pa. These results indicate a more balanced distribution of wall shear stress between the two membrane surfaces.

Among the 16 candidate configurations evaluated, T2820 produced the highest near-wall velocity and wall shear stress but was also accompanied by a high pressure gradient. In comparison, T3823 combined a relatively low pressure gradient with a high near-wall velocity, a pressure-normalized near-wall efficacy index of 1.23, moderate wall shear stress, and improved bilateral shear balance. Based on this overall hydrodynamic balance, T3823 was selected for subsequent flow-field analysis.

### 3.4. Analysis of Local Flow Field Characteristics Induced by the Selected Candidate Configuration

To examine why T3823 was selected by the hydrodynamic screening, its velocity field was compared with that of T-Base in the representative second periodic unit. The streamwise and spanwise coordinates were normalized to [0, 1], and a uniform 40 × 40 statistical grid (1600 equal-area subregions) was used to sample near-wall velocity. This procedure avoids unequal weighting caused by differences in periodic length, fluid volume, or mesh density.

**Figure 6:**
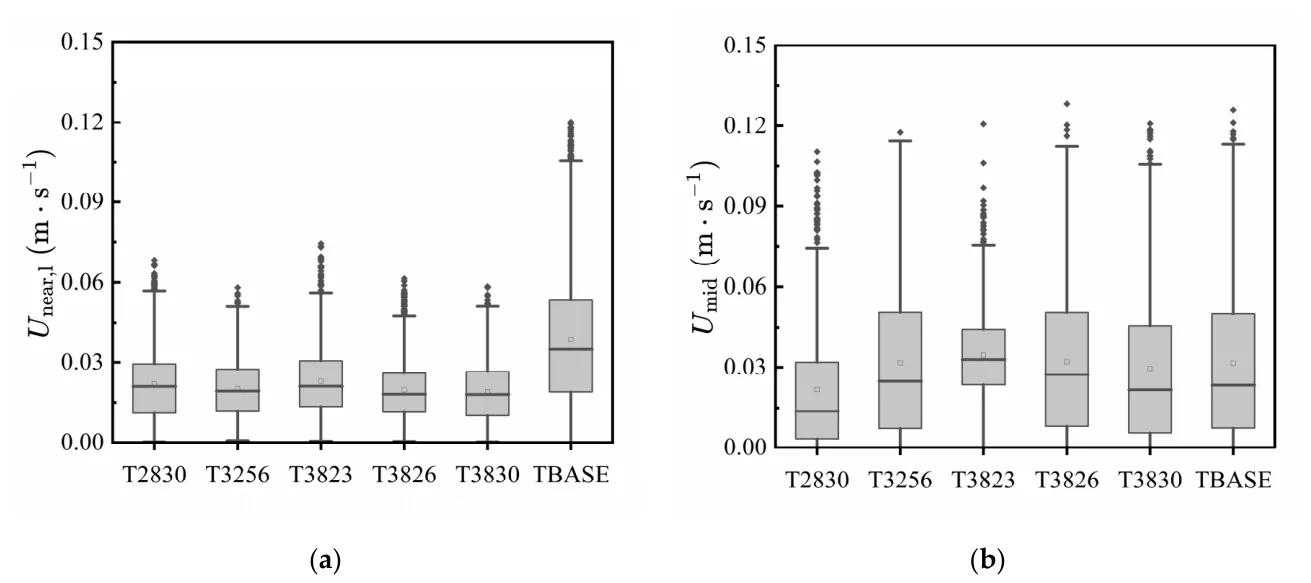
Spatial distributions of velocity magnitude for representative twill-woven spacer configurations: (**a**) near-wall velocity and (**b**) mid-plane velocity obtained from normalized second periodic units.

As shown in Figure 6a, the near-wall velocities of both spacer configurations exhibited significant spatial heterogeneity, which mainly originated from the flow blockage and direction change induced by the periodic crossing of twill-woven filament tows. Compared with T-Base, T3823 more effectively regulated the near-wall velocity distribution, reducing the area of low-velocity regions and increasing the proportion of local high-velocity regions. Specifically, T3823 exhibited a higher median near-wall velocity with a relatively concentrated distribution range (smaller interquartile range), indicating that this configuration promotes tangential flow at the membrane surface and improves the uniformity of the near-wall velocity distribution.

Figure 6b further illustrates the velocity distribution characteristics at the mid-plane cross-section of the channel for both configurations. Owing to the periodic contraction–expansion effect induced by the woven filament tows, the fluid formed continuous acceleration and deceleration regions within the channel. Compared with T-Base, T3823 exhibited a more balanced velocity distribution with a significantly reduced number of low-velocity regions, indicating that it enhanced the fluid perturbation capability while avoiding excessive blockage, which is beneficial for reducing additional hydrodynamic losses.

**Figure 7:**
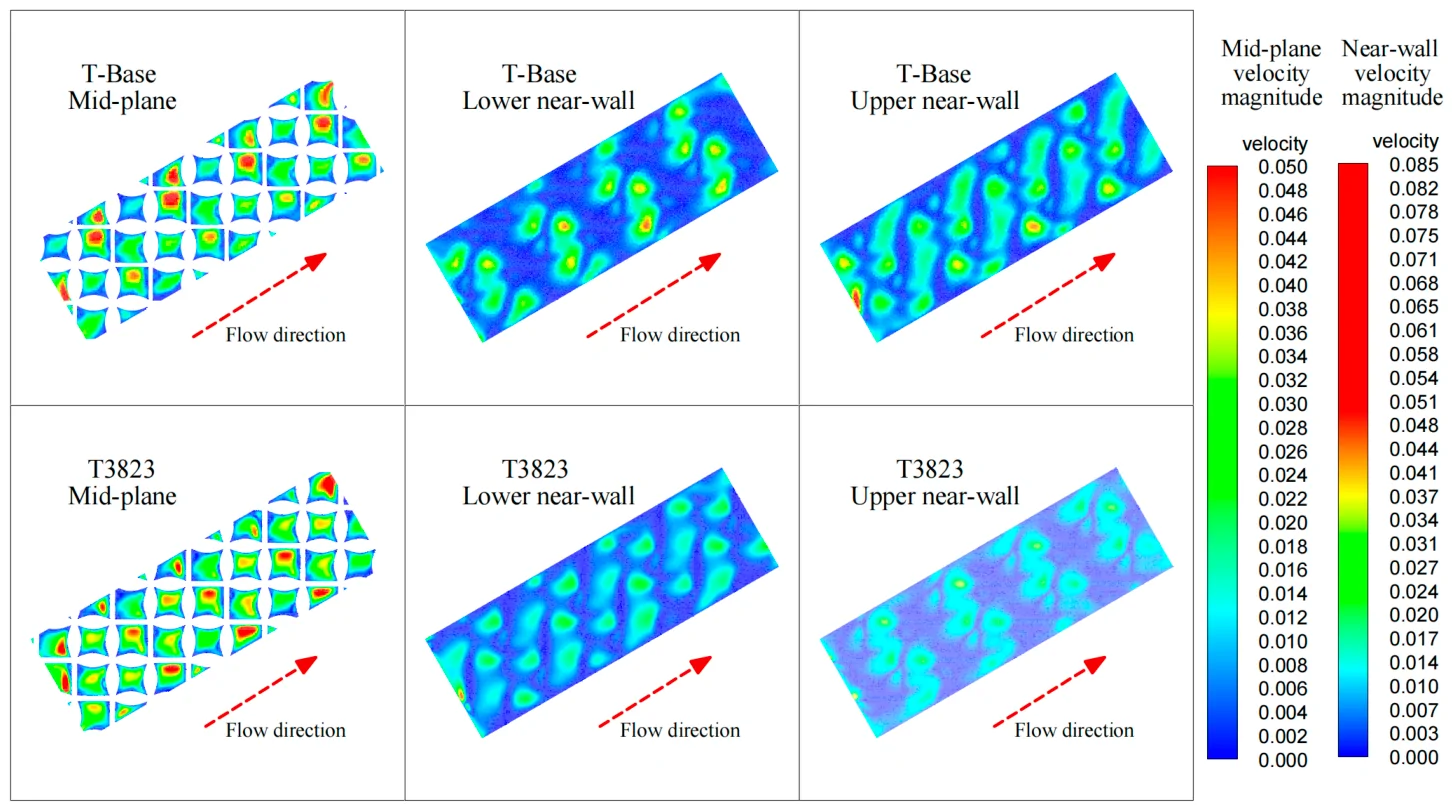
Comparison of velocity-magnitude fields between T-Base and T3823 at the representative second periodic unit: mid-plane and membrane-adjacent regions.

To further observe the local flow structures, Figure 7 presents the velocity contours of T-Base and T3823 at the mid-plane cross-section and in the upper and lower near-membrane regions. At the mid-plane cross-section, both configurations exhibited distinct periodic high-velocity regions, which were mainly generated by local contraction acceleration near the filament crossing positions. However, compared with T-Base, T3823 formed more continuous and uniform high-velocity regions along the flow direction, whereas T-Base exhibited more intense local velocity fluctuations.

Near the upper and lower membrane surfaces, T-Base showed stronger bilateral heterogeneity and more persistent low-velocity regions. Such regions may weaken boundary-layer renewal and could increase deposition risk in an actual filtration system. T3823 produced a more uniform simulated near-wall velocity distribution on both surfaces, but the present pure-water model does not resolve foulant transport or deposition.

The comparative advantage of T3823 therefore lies in a more uniform spatial velocity distribution rather than the highest local peak. By reducing low-velocity regions while maintaining moderate local acceleration, it provided a favorable balance of hydraulic resistance and near-membrane motion within the tested conditions. This hydrodynamic result is potentially relevant to fouling mitigation but is not direct evidence of antifouling performance.

### 3.5. Spatial Distribution Characteristics of Membrane Wall Shear Stress

**Figure 8:**
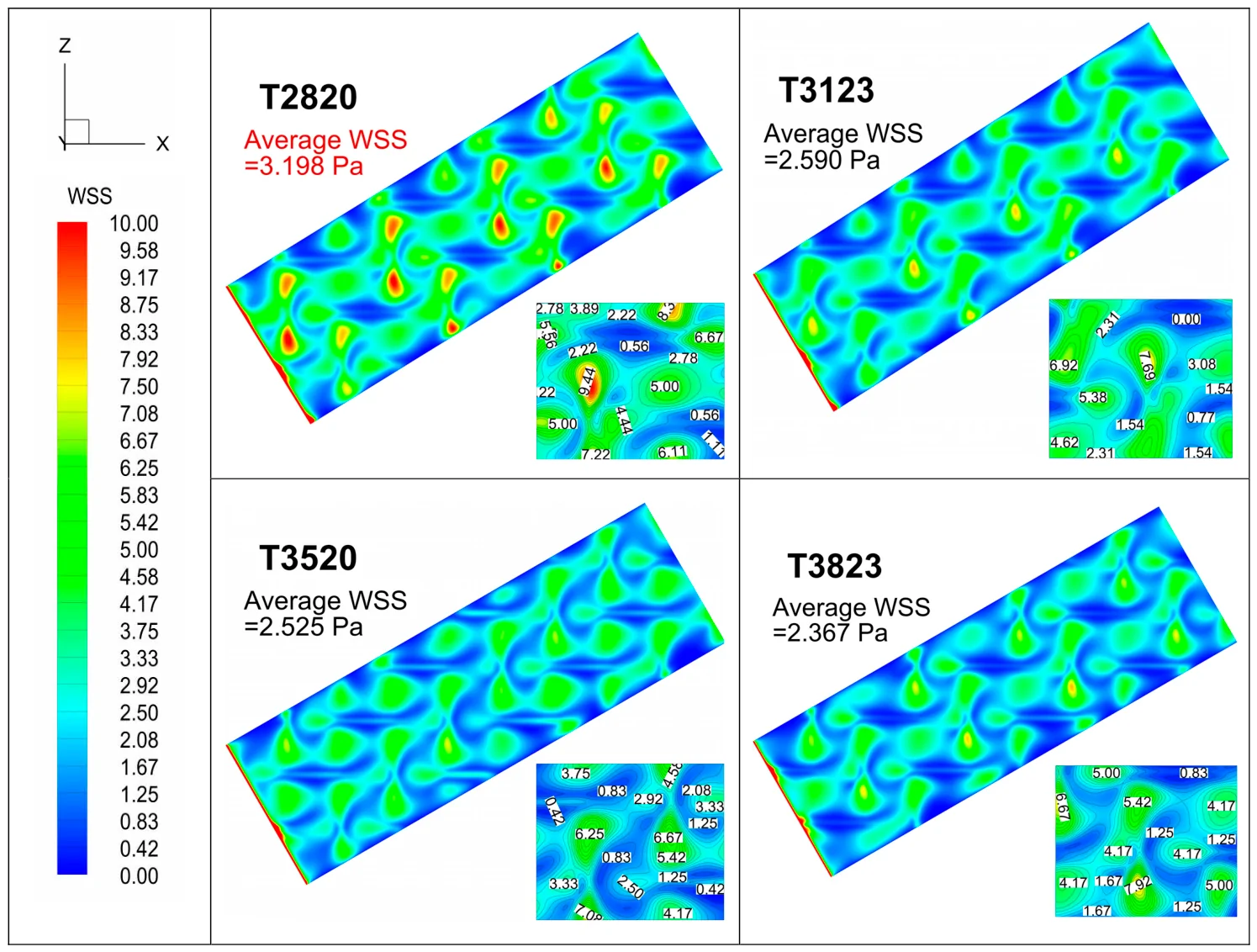
Wall shear stress (WSS) distributions on the lower membrane surface of the representative second periodic unit for four selected twill-woven spacer configurations: T2820, T3123, T3520, and T3823. All panels use a common 0–10 Pa color scale. Labels report lower-membrane area-averaged wall shear stress, not the bilateral mean of the upper- and lower-membrane averages. Wall shear stress distributions for the remaining configurations are provided in the Supplementary Materials.

To further analyze the influence of spacer configurations on the membrane shear environment, the spatial distribution of wall shear stress (WSS) for all spacer configurations was analyzed. As shown in Figure 8, the wall shear stress contours were extracted from the upper and lower membrane surfaces of the representative second periodic unit, and a unified color scale range of 0–10 Pa was employed to ensure direct comparison among different configurations. The average values annotated in the figures represent the area-averaged wall shear stress of the analyzed membrane surfaces.

All spacer configurations exhibited significant spatial heterogeneity of wall shear stress, with periodically alternating high-shear and low-shear regions along the flow direction on the membrane surface. This non-uniform shear distribution mainly originated from the local flow acceleration, direction change, and flow separation effects induced by the periodic crossing of twill-woven filament tows. High-shear regions were generally located at positions where the fluid was squeezed and redirected by the filaments, whereas low-shear regions mainly appeared in the areas after channel expansion. These results indicate that the spacer-induced membrane shear exhibits distinct local characteristics, and a single average value is insufficient to fully describe the actual shear environment on the membrane surface.

Different geometric parameters exerted significant influences on the Wall shear stress distribution. In the T28 series (W = 0.28 mm), with increasing coupled filament diameter–thickness parameter, the average wall shear stress generally exhibited a decreasing trend, from 3.198 Pa for T2820 (D = 0.20 mm) to 2.477 Pa for T2830 (D = 0.30 mm). A similar trend was observed in other filament spacing series: the T31 series (W = 0.31 mm) decreased from 2.566 Pa to 2.315 Pa, the T35 series (W = 0.35 mm) from 2.525 Pa to 2.184 Pa, and the T38 series (W = 0.38 mm) from 2.478 Pa to 2.123 Pa. This trend indicates that although an increase in the coupled filament diameter–thickness parameter can reduce the flow resistance, it simultaneously weakens the local flow perturbation and the membrane shear enhancement capability.

Among the 16 candidates, T2820 exhibited the highest area-averaged wall shear stress (3.198 Pa), along with an extensive high-shear region and substantial hydraulic resistance. In comparison, the T38 configurations exhibited more moderate wall-shear levels. The area-averaged wall shear stress values for T3823, T3826, and T3830 were 2.270, 2.215, and 2.123 Pa, respectively. Thus, T3823 maintained a comparatively high wall-shear level with lower hydraulic resistance than T2820.

Further comparison of the spatial distribution revealed that the advantage of T3823 did not stem from maximizing the local shear intensity, but rather from achieving a more balanced membrane shear distribution. Compared with T-Base, T3823 reduced the continuous low-shear regions and formed more uniformly distributed high-shear regions, indicating that this configuration can improve the momentum transfer efficiency from the bulk fluid to the membrane surface. This result is consistent with the near-wall velocity enhancement phenomenon observed in Section 3.4.

The wall shear stress maps demonstrate that shear magnitude, spatial uniformity, and hydraulic resistance should be considered jointly when evaluating candidate configurations. Among the candidates, T3823 maintained a moderate wall shear stress at the membrane surfaces while exhibiting a lower pressure gradient than the highest-resistance configurations. This balance supports the selection of T3823 for subsequent hydrodynamic analysis.

## 4. Discussion

This study develops a transient laminar model in ANSYS Fluent and validates the model using experimental pure-water pressure gradient data. The present work also conducts sensitivity analyses for computational domain length, mesh resolution, and time step size. Model predictions show great consistency with experimental measurements, with an average relative deviation below 4%. Mesh M2 contains 717,843 cells, and all monitored flow parameters exhibit deviations less than 1.1% between Mesh M2 and Mesh M3. The adopted time step of 0.010 s also delivers time-step-independent results within the test range. Combined with the governing equations, boundary conditions, and initial conditions defined in this work, these validation results fully support the model’s capability for comparative hydrodynamic analysis.

Filament spacing and the coupled parameter of filament diameter and spacer thickness jointly govern the hydrodynamic behaviors of twill-woven spacers. Larger filament spacing increases channel porosity and generally reduces flow resistance. Meanwhile, the coupled diameter-thickness parameter adjusts effective flow passages and modulates local geometric features between filament apexes and the membrane surface. The resulting flow contraction, expansion, redirection, and local flow acceleration explain the non-monotonic variation in pressure drop and near-membrane flow characteristics against individual geometric parameters.

Multi-index evaluation proves that single optimal performance values cannot support reliable spacer candidate screening. Configuration T2820 achieves the maximum near-wall velocity and wall shear stress but induces considerable pressure loss. Configuration T3823 produces a near-wall velocity of Ūnear = 0.0220 m·s^−1^ and a pressure gradient of Gp = 53.5 kPa·m^−1^, with a pressure-normalized near-wall efficiency of $\eta_{U}$ = 1.23. This value reveals that T3823 delivers a 23% higher near-wall velocity per unit pressure gradient than the baseline T-Base configuration. This evaluation index adopts explicit pressure normalization, rather than a simple velocity enhancement ratio.

Bilateral shear balance delivers flow field information that area-averaged shear parameters cannot reflect. Compared with the T-Base configuration, T3823 reduces the upper-to-lower wall shear stress deviation Dτ from 24.91% to 8.30%, achieving a relative reduction of approximately 66.7%. This configuration establishes a more balanced hydrodynamic environment on the membrane surface, while its effects on membrane flux and fouling behavior still require further verification.

Equal-area normalized spatial sampling further confirms that T3823 exhibits a more concentrated near-wall velocity distribution than T-Base, with an interquartile range reduced by 50.7%. Velocity and wall shear stress contour maps reveal that this difference stems from widespread moderate flow across the membrane region, rather than localized isolated high-velocity peaks. Relative to assessments based primarily on surface-averaged quantities or raw CFD-node distributions, the methodological novelty of this study lies in jointly evaluating hydraulic expenditure, near-membrane transport, bilateral wall shear stress balance, and equally weighted spatial distributions. The equal-area sampling procedure also avoids the unequal weighting that can arise when comparing configurations with different periodic lengths and mesh densities.

Overall, T3823 achieved the most favorable balance among hydraulic resistance, near-wall transport, wall shear stress, and bilateral uniformity among the 16 candidate configurations. The proposed multi-indicator, spatially resolved framework provides a systematic basis for the comparative hydrodynamic assessment of twill-woven feed spacers in flat-sheet ultrafiltration cassettes.

## 5. Conclusions

This study combines pure-water pressure-drop measurements with transient three-dimensional simulations in ANSYS Fluent to compare the hydrodynamic performance of a commercial 2/1 twill-woven feed spacer and 16 self-designed candidate geometries. Numerical predictions show good consistency with experimental pressure gradient data, yielding a mean relative deviation below 4%. Evaluations of computational domain length, mesh resolution and time step size confirm numerical independence across all tested ranges. This work explicitly presents the continuity equation, incompressible Navier–Stokes equations, Reynolds number conventions, boundary conditions and initialization procedures to ensure full reproducibility of the modeling framework.

The principal novelty of this study is an experimentally anchored, geometry-resolved screening framework that integrates pressure gradient, near-membrane velocity, wall shear stress, pressure-normalized near-wall efficacy, bilateral wall shear stress balance, and equal-area spatial sampling to identify the most favorable hydrodynamic trade-off within the investigated design space. Under the operating condition of β = 60° and u_s_ = 0.0551 m·s^−1^, T3823 delivers the most favorable hydrodynamic trade-off among the 16 candidates. This configuration achieves a Ū_near_ of 0.0220 m·s^−1^, a Gp of 53.5 kPa·m^−1^, an η_U,i_ of 1.23 and a D_τ_ of 8.30%, alongside a 50.7% lower interquartile range of near-wall velocity compared with T-Base. These findings demonstrate that researchers need to jointly assess local flow uniformity, bilateral shear balance, mean shear characteristics and hydraulic resistance in spacer performance evaluation.

Overall, the proposed multi-indicator, spatially resolved framework provides a systematic approach to the hydrodynamic assessment and design of twill-woven feed spacers in flat-sheet ultrafiltration cassettes. Future work can extend this framework to filtration conditions by combining experiments with representative feed solutions and multiphysics transport modeling.

## Figures and Tables

**Table 1:** Geometric parameters and channel porosity of the investigated twill-woven spacer configurations. Entries are grouped by increasing filament spacing W and, within each W group, by increasing coupled filament-diameter/spacer-thickness level D–H.

Configuration	W (mm)	D (mm)	H (mm)	Channel Porosity, ε (%)
T-Base	0.297	0.215	0.42	70.10
T2820	0.28	0.20	0.38	69.85
T2823	0.28	0.23	0.44	68.38
T2826	0.28	0.26	0.50	65.07
T2830	0.28	0.30	0.58	61.74
T3120	0.31	0.20	0.38	73.69
T3123	0.31	0.23	0.44	70.40
T3126	0.31	0.26	0.50	67.51
T3130	0.31	0.30	0.58	64.23
T3520	0.35	0.20	0.38	76.04
T3523	0.35	0.23	0.44	72.72
T3526	0.35	0.26	0.50	70.30
T3530	0.35	0.30	0.58	66.63
T3820	0.38	0.20	0.38	77.49
T3823	0.38	0.23	0.44	74.28
T3826	0.38	0.26	0.50	71.88
T3830	0.38	0.30	0.58	68.26

**Table 2:** Time-step sensitivity analysis for the selected baseline case.

Δt (s)	Steps	Pressure-Drop Monitor (Pa)	${Ū}_{near}$ ($m\cdot s^{-1}$)	${\bar{\tau }}_{w}$ (Pa)	Relative Deviation (%)
0.020	25	121.4490019	0.044516987	2.799138157	$1.47\times 10^{-5}$
0.010	50	121.4489871	0.044516983	2.799137745	$1.98\times 10^{-10}$
0.005	100	121.4489871	0.044516983	2.799137745	Reference

## Data Availability

The processed hydrodynamic data supporting this study are provided in the Supplementary Materials. The ANSYS Fluent case and data files required to reproduce the reported comparisons are publicly available in the GitHub repository membranes-16-00329-data (release v1.0): https://github.com/uncle-li-007/membranes-16-00329-data/releases/tag/v1.0 (accessed on 17 September 2026).

## Supplementary Materials

The following supporting information can be downloaded at: https://www.mdpi.com/article/10.3390/membranes16100329/s1, Figure S1: Experimental setup for pressure-drop measurement: (a) overall cross-flow circulation system; (b) flat-sheet ultrafiltration cassette with inlet and outlet pressure measurement positions; Table S1: Geometric parameters, mesh information, and channel porosity of investigated spacer configurations; Table S2: Pressure gradient by periodic unit and computational-domain length; Table S3: Mesh sensitivity and deviations from the finest mesh; Table S4: Time-step sensitivity of the archived monitor quantities; Table S5: Experimental and CFD pressure gradients across flow rate and attack angle; Table S6: Definitions used for derived hydrodynamic and distribution metrics; Table S7a: Configuration-level pressure and near-wall velocity metrics; Table S7b: Wall-shear-stress, symmetry, and normalized efficacy metrics; Table S8: Descriptive statistics for node-level attack-angle velocity distributions; Table S9: Recovered spatial-series statistics for six configurations; Table S10: Statistics of the reconstructed normalized 40 × 40 grids; Table S11: Experimental conditions for pressure-drop measurements; Supplementary_Hydrodynamic_Data.xlsx: Processed hydrodynamic data and supporting calculations used in this study.

## Abbreviations

The following abbreviations are used in this manuscript:

CFD	Computational fluid dynamics
