# Supplementary material for: Comparative Hydrodynamic Assessment of Twill-Woven Feed Spacers in Flat-Sheet Ultrafiltration Membrane Cassettes: Balancing Hydraulic Resistance and Near-Membrane Flow Enhancement
Source: Membranes (Basel). 2026 Sep 30;16(10):329. doi: 10.3390/membranes16100329 (PMC13707049; doi:10.3390/membranes16100329)
Supplement: Supplementary file 1 [file membranes-16-00329-s001.zip › membranes-4550818-Supplementary_Materials-UPDATED.pdf]

Comparative Hydrodynamic Assessment of Twill-Woven Feed Spacers in Flat-Sheet Ultrafiltration Membrane Cassettes: Balancing Hydraulic Resistance and Near-Membrane Flow Enhancement

S1. Spacer geometry and case coding

Seventeen twill-woven spacer configurations were investigated in this study, including the commercial reference spacer (T-Base) and 16 geometrically modified configurations. The configuration code TWD represents the corresponding filament spacing W and filament diameter D, where the two numerical digits indicate the nominal dimensions in hundredths of a millimetre. The spacer thickness H was determined by the coupled variation of filament diameter and geometry. The channel porosity was calculated as the ratio between the fluid volume and the total periodic channel envelope volume. Detailed geometric parameters and calculated porosity values for all configurations are summarized in Table S1..

Table S1. Geometric parameters, mesh information, and channel porosity of investigated spacer configurations.

Configuration	Archived angle (°)	W (mm)	D (mm)	H (mm)	Porosity (%)	Cells	Min. OQ
T-Base	0	0.297	0.215	0.420	70.10	717,843	0.257
T2820	60	0.280	0.200	0.380	69.85	564,321	0.080
T2823	60	0.280	0.230	0.440	68.38	701,230	0.081
T2826	60	0.280	0.260	0.500	65.07	839,252	0.085
T2830	60	0.280	0.300	0.580	61.74	1,019,764	0.127
T3120	60	0.310	0.200	0.380	73.69	658,033	0.204
T3123	60	0.310	0.230	0.440	70.40	800,396	0.209
T3126	60	0.310	0.260	0.500	67.51	959,892	0.200
T3130	60	0.310	0.300	0.580	64.23	1,166,140	0.231
T3520	60	0.350	0.200	0.380	76.04	776,955	0.233
T3523	60	0.350	0.230	0.440	72.72	942,257	0.247
T3526	60	0.350	0.260	0.500	70.30	215,248	0.100
T3530	60	0.350	0.300	0.580	66.63	1,355,417	0.213
T3820	60	0.380	0.200	0.380	77.49	875,045	0.223
T3823	60	0.380	0.230	0.440	74.28	1,000,909	0.284
T3826	60	0.380	0.260	0.500	71.88	755,435	0.129
T3830	60	0.380	0.300	0.580	68.26	1,512,187	0.228

S2. Numerical-domain and resolution checks

S2.1 Periodic-domain length

Pressure-gradient values were extracted for each periodic unit from computational domains containing two to five repeating units. The first periodic unit exhibited a higher pressure gradient due to the influence of inlet-flow development, whereas the downstream units gradually approached a stable periodic state. Based on the reduction of period-to-period variation, the second periodic unit was selected as the representative evaluation region in the manuscript. The three-period computational domain was used to ensure that the analyzed region was sufficiently separated from the inlet boundary while maintaining computational efficiency.

Table S2. Pressure gradient by periodic unit and computational-domain length

Domain length (periods)	Unit no.	G_p (MPa•m⁻¹)	G_p (kPa•m⁻¹)	Interpretation
2	1	0.06713	67.132	Inlet-development unit
2	2	0.02892	28.920	Post-inlet unit
3	1	0.05060	50.596	Inlet-development unit
3	2	0.02726	27.262	Post-inlet unit
3	3	0.02762	27.621	Periodic/post-inlet unit
4	1	0.05696	56.963	Inlet-development unit
4	2	0.02765	27.655	Post-inlet unit
4	3	0.02758	27.575	Periodic/post-inlet unit
4	4	0.02773	27.734	Periodic/post-inlet unit
5	1	0.05236	52.357	Inlet-development unit
5	2	0.02675	26.754	Post-inlet unit
5	3	0.02680	26.800	Periodic/post-inlet unit
5	4	0.02688	26.876	Periodic/post-inlet unit
5	5	0.02696	26.955	Periodic/post-inlet unit

S2.2 Mesh-independence assessment

Four mesh resolutions were evaluated based on five hydrodynamic quantities, including pressure drop, upper- and lower-membrane near-wall velocities, and upper- and lower-membrane wall shear stresses (WSS). Relative deviations were calculated using the finest mesh (M3) as the reference case. Although M3 provided the highest spatial resolution, the M2 mesh was selected for subsequent simulations because the predicted values of all monitored quantities showed only small deviations of 0.56–1.06% compared with M3, while requiring substantially lower computational cost.

Table S3. Mesh sensitivity and deviations from the finest mesh

Mesh	Cells	Max size (mm)	U lower	U upper	WSS upper	WSS lower	Δp_2 (Pa)	Max dev. (%)
M0-BL	168,192	0.072	0.011136	0.015305	1.1178	1.4321	44.750	73.84
M1	284,300	0.02 mm	0.011368	0.008529	1.1261	1.4433	45.173	5.38
M2	717,843	0.016	0.011761	0.008755	1.1576	1.4871	47.449	1.06
M3	1,184,521	0.016	0.011830	0.008804	1.1700	1.5015	47.743	0.00

S2.3 Time-step sensitivity

Three time steps were evaluated over a common simulated duration. The 0.010 s and 0.005 s cases are numerically identical at the displayed precision, supporting selection of $\Delta t = 0.010$ s.

Table S4. Time-step sensitivity of the archived monitor quantities

Δt (s)	Steps	Pressure monitor (Pa)	Near-wall metric ($m \cdot s^{-1}$)	WSS metric (Pa)	Role
0.020	25	121.4490019	0.044516987	2.799138157	Comparison
0.010	50	121.4489871	0.044516983	2.799137745	Selected
0.005	100	121.4489871	0.044516983	2.799137745	Comparison

S3. Experimental validation of pressure gradients

The compiled validation dataset compares experimentally measured pressure gradients with CFD predictions at six flow attack angles β. The main manuscript presents the validation results within the flow-rate range of 350–540 mL·min^{-1}. Additional measurements at 550 and 580 mL·min^{-1}, available from the original experimental dataset, are retained in the Supplementary Materials and are explicitly indicated as supplementary data points outside the primary plotted range..

Table S5. Experimental and CFD pressure gradients across flow rate and attack angle

Flow (mL min^{-1})	Experiment	CFD 0°	CFD 15°	CFD 30°	CFD 45°	CFD 60°	CFD 75°
350	0.07265	0.02693	0.06146	0.07455	0.07413	0.07485	0.06067
400	0.08382	0.03132	0.07213	0.08739	0.08696	0.08775	0.07108
460	0.10618	0.03673	0.08536	0.10331	0.10294	0.10377	0.08396
500	0.11735	0.04046	0.09461	0.11440	0.11411	0.11492	0.09290
540	0.12853	0.04434	0.10429	0.12602	0.12584	0.12660	0.10225
550	0.15647	0.04526	0.10657	0.12876	0.12861	0.12936	0.10445
580	0.16765	0.04825	0.11405	0.13774	0.13771	0.13840	0.11167

S4. Definitions and calculation rules

The following calculation procedures ensure the reproducibility of all derived quantities provided in the supplementary workbook. Original input parameters are retained in dedicated columns, and derived values are calculated using embedded Excel formulas wherever applicable.

Table S6. Definitions used for derived hydrodynamic and distribution metrics

Quantity	Definition	Interpretation
Channel porosity	$\varepsilon = \frac{\mathrm{Vfluid}}{\mathrm{Vchannel}} \times 100\%$	Fluid volume divided by the periodic channel-envelope volume.
Pressure gradient	$G_p = \frac{\Delta p_2}{L_2}$	Δp_2 in Pa and L_2 in mm give G_p numerically in kPa m^{-1}.
Mean near-wall velocity	$\bar{U} = \frac{U_{upper} + U_{lower}}{2}$	Arithmetic mean of the upper and lower near-membrane planes.
Mean WSS	$\bar{\tau} = \frac{\tau_{upper} + \tau_{lower}}{2}$	Arithmetic mean of area-averaged membrane-surface WSS.
Bilateral WSS deviation	$D_\tau = \frac{\lvert\tau_{upper} - \tau_{lower}\rvert}{\bar{\tau}} \times 100\%$	Lower values indicate more symmetric shear loading.
Velocity enhancement	$E_U = \frac{\bar{U}}{\bar{U}_{ref}}$	Unnormalized mean-velocity ratio.
Pressure-normalized efficacy	$\eta_U = \frac{\bar{U} / \bar{U}_{ref}}{G_p / G_{p,ref}}$	Hydrodynamic efficacy per unit pressure-gradient increase; not a simple velocity ratio.
Pressure-normalized WSS efficacy	$\eta_\tau = \frac{\bar{\tau} / \bar{\tau}_{ref}}{G_p / G_{P,ref}}$	Mean-WSS response per unit pressure-gradient increase.
Normalized equal-area grid	$n^* = \frac{n - n_{min}}{n_{max} - n_{min}}$	Each plane is divided into 40 × 40 bins; occupied-bin means are preserved and empty-bin handling is declared.

S5. Complete hydrodynamic performance matrix

The complete dataset covering all 17 spacer configurations is divided into two tables for clarity. Table S7a summarizes the geometric parameters, hydraulic performance, and velocity-related quantities, whereas Table S7b presents the wall shear stress (WSS), bilateral membrane shear balance, and dimensionless evaluation indices. The original input parameters and calculation procedures, including inlet and outlet pressure values, velocity data, and derived quantities, are preserved in the Excel worksheet “S6_Hydrodynamics” to ensure data traceability and reproducibility.

Table S7a. Configuration-level pressure and near-wall velocity metrics

Config.	Angle (°)	ε (%)	Δp_2 (Pa)	L_2 (mm)	G_p (kPa m^{-1})	U lower	U upper	$\bar{U}$ (m s^{-1})
T-Base	0	70.10	47.449	1.536	30.891	0.011761	0.008755	0.010258
T2820	60	69.85	141.721	1.440	98.417	0.024378	0.021883	0.023131

Config.	Angle (°)	ε (%)	Δp_2 (Pa)	L_2 (mm)	G_p (kPa m^{-1})	U lower	U upper	$\bar{U}$ (m s^{-1})
T2823	60	68.38	112.308	1.530	73.404	0.021121	0.018857	0.019989
T2826	60	65.07	105.869	1.620	65.351	0.020476	0.018087	0.019282
T2830	60	61.74	101.106	1.740	58.107	0.019424	0.017140	0.018282
T3120	60	73.69	112.088	1.530	73.260	0.021593	0.019165	0.020379
T3123	60	70.40	108.082	1.620	66.717	0.020187	0.018104	0.019146
T3126	60	67.51	103.719	1.710	60.654	0.019251	0.017254	0.018253
T3130	60	64.23	98.603	1.830	53.882	0.018230	0.016257	0.017243
T3520	60	76.04	102.030	1.650	61.837	0.019677	0.017222	0.018449
T3523	60	72.72	103.179	1.740	59.298	0.019124	0.017165	0.018145
T3526	60	70.30	93.740	1.830	51.224	0.017126	0.015449	0.016288
T3530	60	66.63	94.214	1.950	48.315	0.017295	0.015479	0.016387
T3820	60	77.49	105.423	1.740	60.588	0.019420	0.017395	0.018408
T3823	60	74.28	98.254	1.830	53.690	0.023309	0.020709	0.022009
T3826	60	71.88	90.036	1.920	46.894	0.017805	0.015747	0.016776
T3830	60	68.26	91.018	2.040	44.616	0.016729	0.014964	0.015846

Table S7b. Wall-shear-stress, symmetry, and normalized efficacy metrics

Config.	WSS upper	WSS lower	WSS mean	Dτ (%)	$G_p/G_{p,ref}$	$\bar{U}/\bar{U}_{ref}$	$\bar{\tau}/\bar{\tau}_{ref}$	ηU	ητ
T-Base	1.1576	1.4871	1.3224	24.91	1.000	1.000	1.000	1.000	1.000
T2820	2.9546	3.1977	3.0761	7.90	3.186	2.255	2.326	0.708	0.730
T2823	2.7258	2.5046	2.6152	8.46	2.376	1.949	1.978	0.820	0.832
T2826	2.4019	2.6268	2.5144	8.95	2.116	1.880	1.901	0.888	0.899
T2830	2.2607	2.4767	2.3687	9.12	1.881	1.782	1.791	0.947	0.952
T3120	2.7853	2.5657	2.6755	8.21	2.372	1.987	2.023	0.838	0.853
T3123	2.3881	2.5903	2.4892	8.12	2.160	1.866	1.882	0.864	0.872
T3126	2.2646	2.4589	2.3617	8.23	1.963	1.779	1.786	0.906	0.910
T3130	2.1211	2.3155	2.2183	8.76	1.744	1.681	1.678	0.964	0.962
T3520	2.2627	2.5253	2.3940	10.97	2.002	1.799	1.810	0.898	0.904
T3523	2.2477	2.4386	2.3431	8.14	1.920	1.769	1.772	0.921	0.923
T3526	2.0716	2.2569	2.1642	8.56	1.658	1.588	1.637	0.958	0.987
T3530	2.0067	2.1837	2.0952	8.45	1.564	1.597	1.584	1.021	1.013
T3820	2.2783	2.4776	2.3780	8.38	1.961	1.794	1.798	0.915	0.917
T3823	2.1783	2.3669	2.2726	8.30	1.738	2.146	1.719	1.234	0.989
T3826	2.0309	2.2153	2.1231	8.68	1.518	1.635	1.606	1.077	1.058
T3830	1.9304	2.1027	2.0166	8.55	1.444	1.545	1.525	1.070	1.056

S6. Attack-angle and spatial-distribution statistics

S6.1 Attack-angle distributions

Node-level velocity samples were extracted from the near-membrane regions for six flow attack angles β. The supplementary workbook contains all 75,311 velocity data points in the worksheet "Raw_Angle" and provides the corresponding descriptive statistical analysis in "S7_Angle_Stats".

Table S8. Descriptive statistics for node-level attack-angle velocity distributions

Angle (°)	Plane	n	Mean	SD	Q1	Median	Q3	IQR	CV (%)
0	membrane-wall	21,690	0.022201	0.019448	0.006553	0.016432	0.033484	0.026931	87.6
0	near-wall	25,246	0.016499	0.014954	0.005475	0.010288	0.023437	0.017962	90.6
15	membrane-wall	20,379	0.033613	0.031064	0.008990	0.024081	0.049480	0.040489	92.4
15	near-wall	23,039	0.026531	0.023278	0.008655	0.019502	0.036834	0.028179	87.7
30	membrane-wall	23,040	0.034105	0.026252	0.012909	0.028507	0.049591	0.036682	77.0
30	near-wall	23,041	0.029788	0.022889	0.009963	0.025250	0.043548	0.033585	76.8
45	membrane-wall	31,055	0.035030	0.027723	0.012425	0.029061	0.051445	0.039020	79.1
45	near-wall	65,535	0.029913	0.017979	0.016066	0.028170	0.041785	0.025719	60.1
60	membrane-wall	23,042	0.033923	0.026135	0.012901	0.028636	0.049239	0.036337	77.0
60	near-wall	23,043	0.034010	0.023222	0.014474	0.031557	0.049090	0.034616	68.3
75	membrane-wall	23,044	0.032013	0.027264	0.009172	0.026115	0.048033	0.038861	85.2
75	near-wall	75,311	0.029415	0.017714	0.015718	0.027793	0.040605	0.024888	60.2

S6.2 Selected-model spatial statistics and 40 × 40 normalization

Six configuration-specific datasets containing processed near-membrane and mid-plane velocity distributions were prepared for statistical analysis. For T-Base and T3823, coordinate-resolved node data were further mapped onto a common normalized 40 ×40 spatial grid. This procedure provides an equal-area spatial representation for direct comparison while preserving the number of occupied bins, empty bins, and the original spatial sampling information..

Table S9. Recovered spatial-series statistics for six configurations

Config.	Plane	n	Mean	SD	Q1	Median	Q3	IQR	CV (%)
T-Base	near-wall	20,857	0.035374	0.027181	0.013390	0.031449	0.051147	0.037758	76.8
T-Base	mid-plane	19,323	0.030236	0.027955	0.007011	0.023459	0.046719	0.039709	92.5
T2830	near-wall	59,113	0.019020	0.013909	0.007172	0.017726	0.027560	0.020388	73.1
T2830	mid-plane	33,684	0.023777	0.025107	0.005082	0.016097	0.034248	0.029166	105.6
T3526	near-wall	15,339	0.016474	0.012890	0.005600	0.014602	0.024100	0.018501	78.2
T3526	mid-plane	22,111	0.030885	0.027056	0.008453	0.025133	0.047301	0.038848	87.6

Config.	Plane	n	Mean	SD	Q1	Median	Q3	IQR	CV (%)
T3823	near-wall	96,249	0.022151	0.014125	0.012081	0.020414	0.030406	0.018325	63.8
T3823	mid-plane	44,062	0.034346	0.028465	0.010850	0.028654	0.052823	0.041973	82.9
T3826	near-wall	12,695	0.014784	0.012427	0.004487	0.012239	0.021587	0.017100	84.1
T3826	mid-plane	40,609	0.036240	0.028009	0.014171	0.031464	0.052856	0.038685	77.3
T3830	near-wall	80,528	0.016652	0.012248	0.006356	0.015500	0.023976	0.017620	73.6
T3830	mid-plane	49,149	0.029771	0.027226	0.007218	0.022891	0.044929	0.037711	91.4

Table S10. Statistics of the reconstructed normalized 40 × 40 grids

Config.	Plane	Grid convention	Source nodes	Occupie d	Empty	Mean	IQR
T-Base	near-wall	40x40 occupied-bin means	6,842	1529	71	0.038606	0.034709
T-Base	near-wall	40x40 all bins, empty=0	6,842	1529	71	0.036893	0.036666
T-Base	mid-plane	40x40 occupied-bin means	5,931	1002	598	0.031597	0.042637
T-Base	mid-plane	40x40 all bins, empty=0	5,931	1002	598	0.019788	0.033701
T3823	near-wall	40x40 occupied-bin means	31,868	1600	0	0.022891	0.017502
T3823	near-wall	40x40 all bins, empty=0	31,868	1600	0	0.022891	0.017502
T3823	mid-plane	40x40 occupied-bin means	22,276	1578	22	0.034350	0.020966
T3823	mid-plane	40x40 all bins, empty=0	22,276	1578	22	0.033878	0.021216

S7.Experimental setup for pressure-drop measurement

A laboratory-scale cross-flow circulation system was used for the experimental measurement of pressure drop in the spacer-filled flat-sheet ultrafiltration cassette. As shown in Figure S1, the setup consisted of a feed reservoir, circulation pump, flow-control valves, pressure gauges, connecting tubing, and a flat-sheet membrane cassette. The twill-woven feed spacer was installed inside the cassette during testing. Deionized water was circulated through the channel, and the inlet and outlet pressures were measured using pressure gauges installed before and after the membrane cassette.

The pressure drop across the spacer-filled channel was calculated from the difference between the inlet and outlet pressures. The flow rate was adjusted using the control valve, and pressure values were recorded after reaching a stable operating condition. The experimental pressure-drop data were used for CFD model validation.

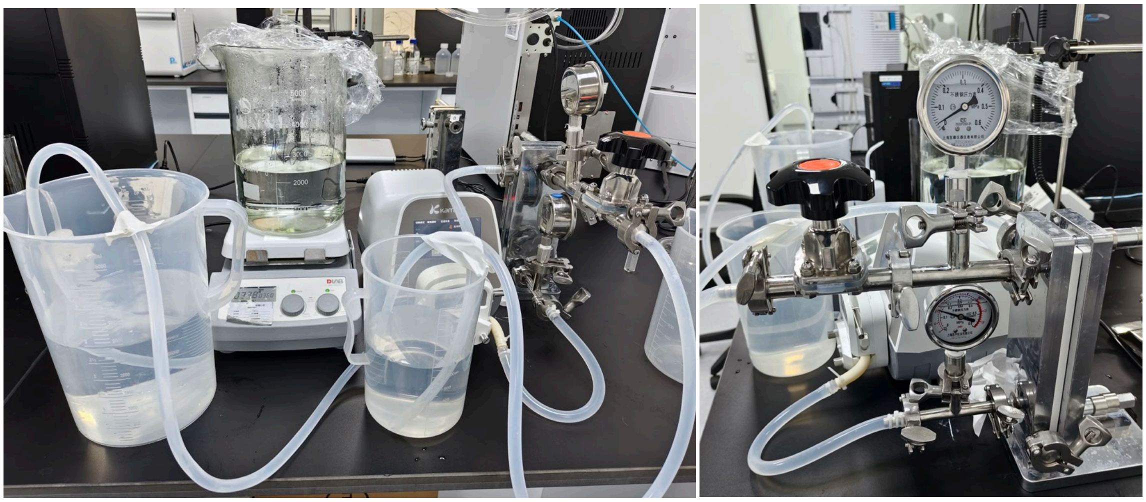

Figure S1.Experimental setup for pressure-drop measurement: (a) overall cross-flow circulation system; (b) flat-sheet ultrafiltration cassette with inlet and outlet pressure measurement positions.

Table S11. Experimental conditions for pressure-drop measurements

Parameter	Value
Testing fluid	Deionized water
Membrane module	Flat-sheet ultrafiltration cassette
Spacer type	2/1 twill-woven spacer
Flow mode	Cross-flow circulation
Measured parameter	Pressure drop
Pressure measurement	Inlet/outlet pressure difference
